# Optimising migraine treatment: from drug-drug interactions to personalized medicine

**DOI:** 10.1186/s10194-019-1010-3

**Published:** 2019-05-17

**Authors:** Leda Marina Pomes, Martina Guglielmetti, Enrico Bertamino, Maurizio Simmaco, Marina Borro, Paolo Martelletti

**Affiliations:** 1 Residency Program in Laboratory Medicine, Gabriele d’Annunzio University, Chieti, Italy; 2 Regional Referral Headache Centre, Sant’Andrea Hospital, Rome, Italy; 3 Department of Medical, Surgical and Experimental Sciences, University of Sassari, Sassari, Italy; 4 Residency Program in Hygiene and Preventive Medicine, Sapienza University of Rome, Rome, Italy; 5 Department of Neurosciences, Mental Health and Sensory Organs, Sapienza University of Rome, Rome, Italy; 6 Department of Clinical and Molecular Medicine, Sapienza University of Rome, Rome, Italy; 7 Internal Medicine and Emergency Medicine Unit, Sant’Andrea Hospital, Rome, Italy

**Keywords:** Personalized medicine, Pharmacogenomic, Anti-migraine drugs, Polytherapies, Gepants, Ditans, CGRP monoclonal antibodies

## Abstract

Migraine is the most disabling and expensive chronic disorders, the etiology of which is still not fully known. The neuronal systems, (glutammatergic, dopaminergic, serotoninergic and GABA-ergic) whose functionality is partly attributable to genetically determined factors, has been suggested to play an important role. The treatment of acute attacks and the prophylactic management of chronic forms include the use of different category of drugs, and it is demonstrated that not each subject has the same clinical answer to them. The reason of this is to be searched in different functional capacity and quantity of phase I enzymes (such as different isoforms of CYP P450), phase II enzymes (such as UDP-glucuronosyltransferases), receptors (such as OPRM1 for opioids) and transporters (such as ABCB1) involved in the metabolic destiny of each drug, all of these dictated by DNA and RNA variations. The general picture is further exacerbated by the need for polytherapies, often also to treat comorbidities, which may interfere with the pharmacological action of anti-migraine drugs. Personalized medicine has the objective of setting the optimal therapies in the light of the functional biochemical asset and of the comorbidities of the individual patient, in order to obtain the best clinical response. Novel therapeutic perspectives in migraine includes biotechnological drugs directed against molecules (such as CGRP and its receptor) that cause vasodilatation at the peripheral level of the meningeal blood vessels and reflex stimulation of the parasympathetic system. Drug-drug interactions and the possible competitive metabolic destiny should be studied by the application of pharmacogenomics in large scale. Drug-drug interactions and their possible competitive metabolic destiny should be studied by the application of pharmacogenomics in large scale.

## Introduction

According with World Health Report in 2001, migraine is the most disabling and expensive Chronic disorders [1] representing the major cause of non-fatal disease – related disability [2].

Migraine is a common disorder connoted by recurrent headache attacks with nausea, vomiting, hyper sensibility to light, sound and smell (defined as Migraine without aura, MO) and, in 25% of cases, neurological symptoms (defined as Migraine with aura, MA) [3].

The disorder is more frequent in female (3,1 = F:M) with a peak of prevalence between ages of 22 and 55 years old [4].

Genetic factors have been implicated in many aspects of migraine: the aetiology, the tendency to become chronic, the sensitivity to pharmacological treatment. The last aspect offers the possibility to design personalized treatments in order to achieve improved therapeutic success.

### Genetic roots of migraine

Glutammatergic, dopaminergic, serotoninergic and GABA-ergic systems are implicated in the Migraine Headache etiology. Genetic variations affecting expression in terms of quality and quantity of proteins, enzymes, receptors and channels belonging to these systems have been widely described [5–7] and the genetic component of the disease is estimate around a 50%.

Linkage analysis and genome-wide association studies (GWAS) have been conducted on patients with common migraine. However, linkage analyses have minimal power of detection when studying genetic bases of complex traits and multifactorial disease such migraine (not showing a simple Mendelian pattern of transmission), and most results proved to be “false” positive, failing to be replicated in larger cohorts or being contradictory. Differently, GWAS are based on genome-wide data mining on automatic array platforms in which hundreds of thousand SNPs are queried and showed a high power to detect common variants related to migraine [6]. Among these, some are involved specifically in the susceptibility to the development of the pathology [8, 9], as polymorphisms in the encoding endothelin type A receptor (EDNRA), methylenetetrahydrofolate reductase (MHTFR), endothelial nitric oxide synthase (NOS3), angiotensin-converting enzyme (ACE), β-2 transforming growth factor (TGFB2) and its receptor (TGFB2R), neurogenic locus notch homolog protein 3 (NOTCH3).

Therapeutic failure could be traced back to the use of drugs undergoing non-optimal metabolism in a specific patient. Treatment failure can in turn lead to overuse of acute medication, often without great results. Overuse of acute medication is commonly identified as the most important risk factors for chronic headache (CH, group of headaches occurring daily or almost daily) and a causative factor for medications overuse headache (MOH) [10]. About the genetic liability of this last form of complication of migraine (MOH), such as for the common ones’, an involvement of some polymorphisms of 5HTT (such as the 5-HTTLPR) [11, 12] has been hypothesized. Moreover, drug dependence has been associated to polymorphism in genes regulating monoaminergic transmission [13].

### Pharmacogenomics

The fact that only the 50% of migraine patients adequately respond to acute and prophylaxis therapies suggest that migraine patients react differently to given drugs [14]. The patient’s response (efficacy and toxicity) to a drug is affected by DNA and RNA variations in that patient, resulting in different rates of therapeutic effect as in different risk of adverse events, also burdening the health expenses [15–17].

The genomic characterization of the allelic variants carried by the patients allows identification of drug-interacting proteins (metabolic enzymes, transporters, targets) with an altered activity. Since alteration of the drug-protein interactions can change both the pharmacokinetic and pharmacodynamic profiles of the administered drug, recognition of such alteration may be used to avoid administration of non-appropriate drugs, choosing an alternative medication in the same pharmacological class.

Moreover, in the next future it will be possible to design new drugs targeted on a patient’s genetic trait.

By cross-referencing the data relating to each drug used in a politreated patient, it is possible to predict drug-drug interactions using web-based knowledgebases. The same interactions impact differently on the metabolic destiny of each of the other drugs included in the therapy, so it is possible, in light of the patient’s genomic profile, to optimize the therapeutic choices by entrusting treatment to drugs that do not interfere with each other and do not interfere with the profile of the patient in question.

Many drugs are metabolized by isoforms of Cytochrome P450, membrane-associated proteins in the endoplasmic reticulum [18], and different studies show as they are particularly important in drugs used in migraine therapy.

Here we consider the most frequent pharmacological classes used in the treatment of migraine attacks such as NSAIDs, triptans and opioids, moreover we consider tricyclic antidepressants most used in prophylactic therapy [19].

#### NSAIDs

NSAIDs represent the most frequent drug’s class used by migraine sufferers (with at first place Ketoprofen, used in 41% of cases in migraine attack) [19].

This medications’ metabolism depends on the phase I metabolic enzymes CYP P450, in particular CYP2C9 and CYP2C8 and frequently on the phase II metabolic enzyme UDP-glucuronosyltransferases [20, 21].

Among the SNPs indentified in the CYP2C9 gene, the *2 (rs1799853) and the *3 (rs1057910), coding for a change of amino-acid sequence, are those associated with significant reductions of enzyme activity [22, 23].

Approximately 35% of the human total CYP2C-encoded enzymes in the liver belong to the CYP2C8 subfamily [24]. Among the 16 allelic variants of CYP2C8, the *2, and *5 are clinically the most important [25], but also the *3 and the *4 are often detected, also if with different frequencies between races.

**Fig. 1:**
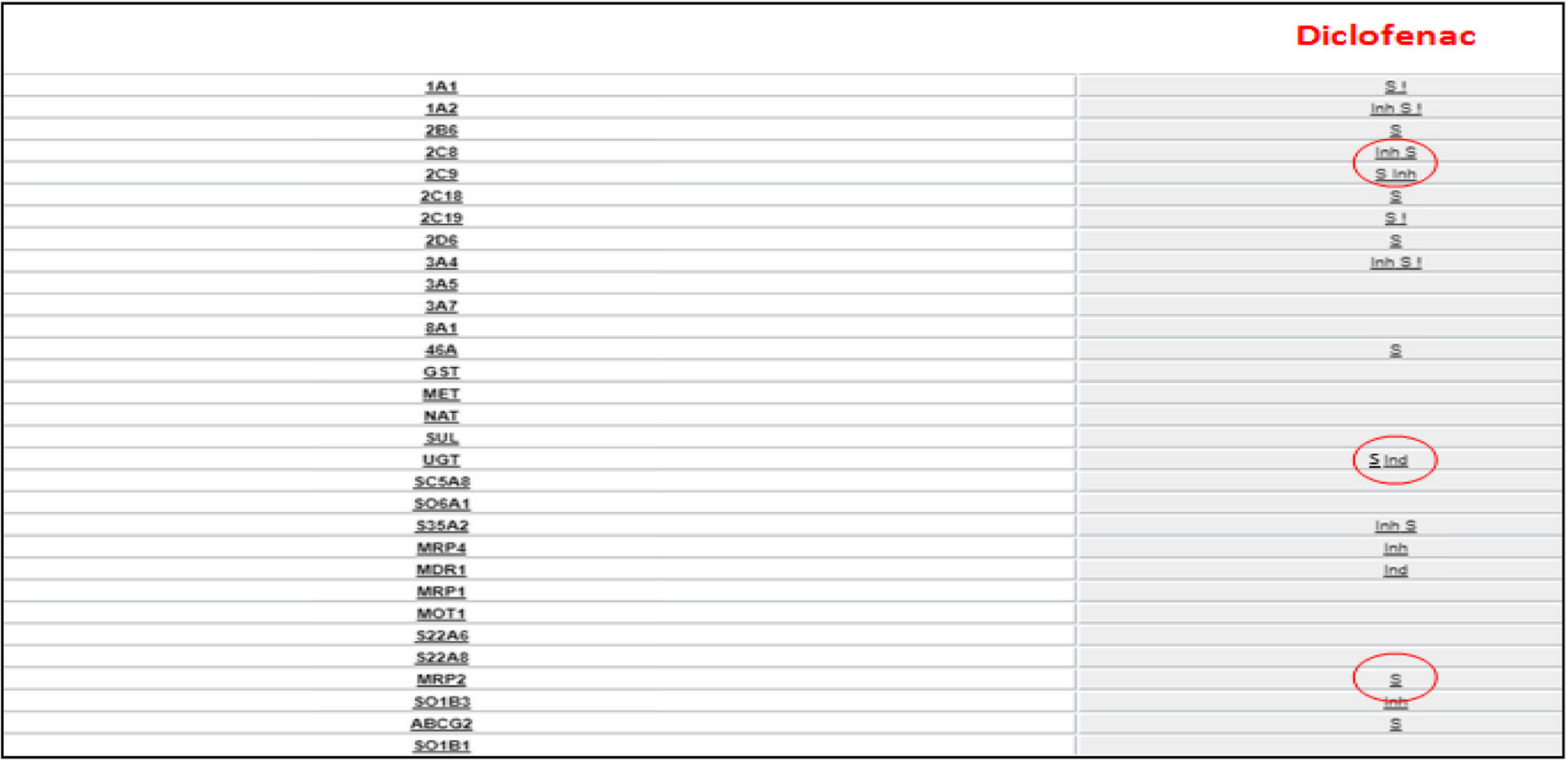
Diclofenac metabolic profile. In the left column there is the list of drug metabolizing enzymes and drug transporters, one for each row; in the right column relationship between corresponding transporter or enzyme of the row and diclofenac: is indicated by the symbol ‘S’ for substrate, ‘Inh’ for inhibitor and ‘Ind’ for inducer. Enzymes CYP 2C9, CYP2C8 and UGT and transporter MRP2 (ABCC2) are rimmed to emphasize their importance in diclofenac’s metabolic destiny. Related page at the website http://bioinformatics.charite.de/transformer

**Fig. 2:**
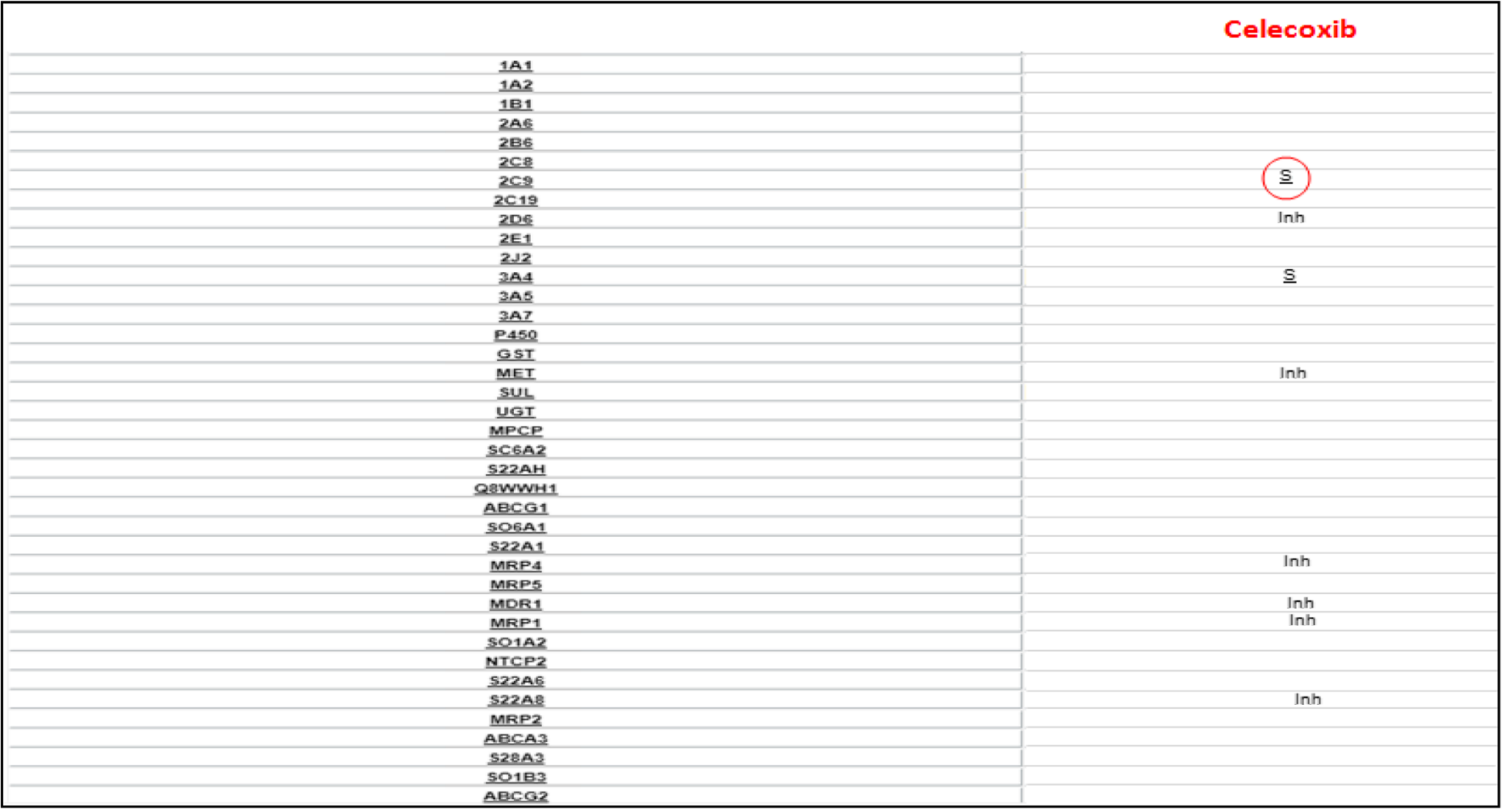
Celecoxib metabolic profile. In the left column there is the list of drug metabolizing enzymes and drug transporters, one for each row; in the right column relationship between corresponding transporter or enzyme of the row and celecoxib: is indicated by the symbol ‘S’ for substrate and ‘Inh’ for inhibitor. Enzyme CYP 2C9 is rimmed to emphasize their importance in celecoxib’s metabolic destiny. Related page at the website http://bioinformatics.charite.de/transformer

In patients carriers of these variants a reduction in therapeutic efficacy (by reducing metabolism or clearance), and an increase in dose-dependent adverse effects [26], are frequent, i.e. CYP2C8*3, CYP2C9*2, *3 and UGT2B7 coding for a low-activity enzyme are implicated in the hepatotoxic effects of Diclofenac [25, 27] [Fig. 1], whereas the loss of function allele CYP2C9*3, is associated to a reduction of celecoxib clearance compared to the wild type [28] [Fig. 2].

**Fig. 3:**
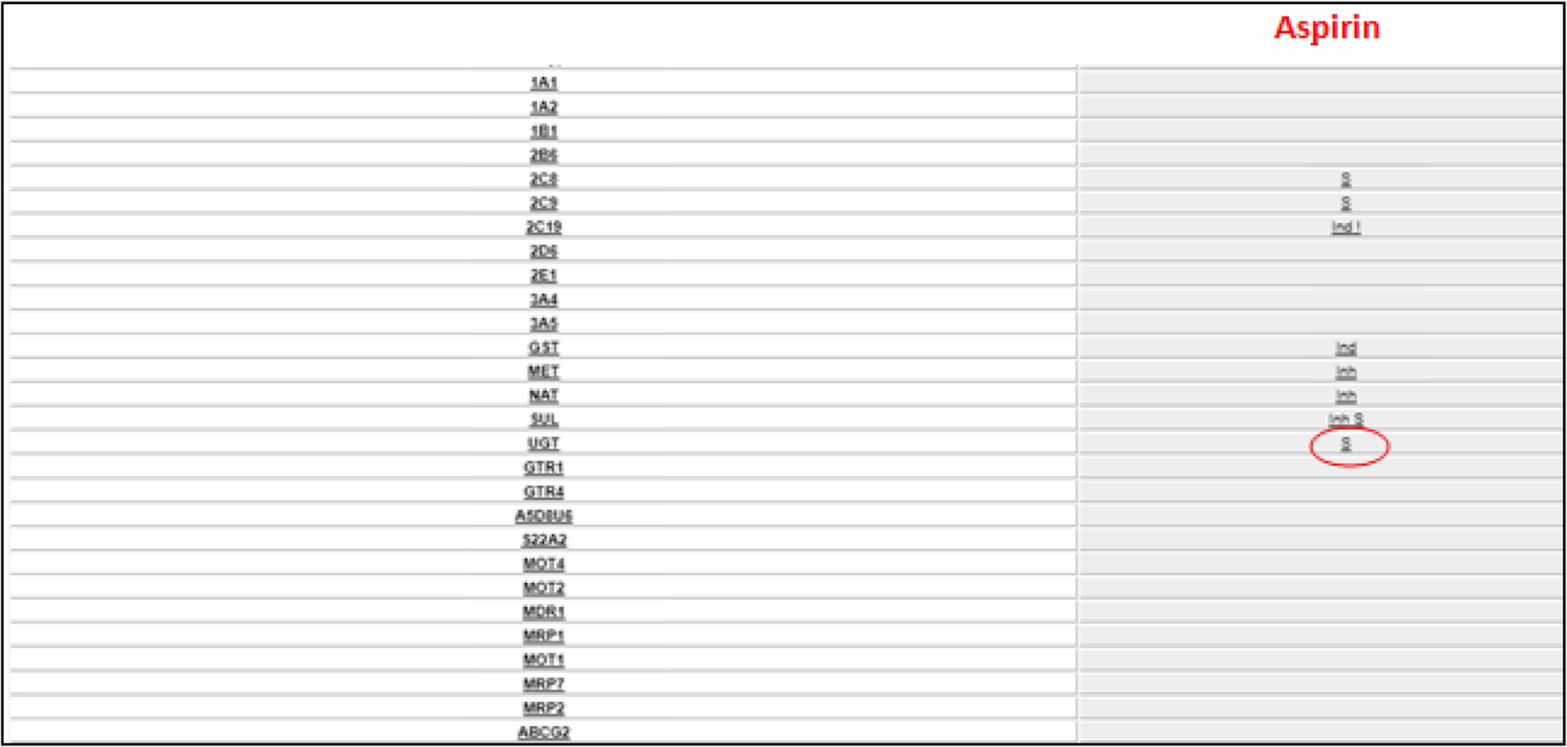
Aspirin metabolic profile. In the left column there is the list of drug metabolizing enzymes and drug transporters, one for each row; in the right column relationship between corresponding transporter or enzyme of the row and aspirin: is indicated by the symbol ‘S’ for substrate, ‘Inh’ for inhibitor and ‘Ind’ for inducer. Enzyme UGT is rimmed to emphasize their importance in aspirin’s metabolic destiny. Related page at the website http://bioinformatics.charite.de/transformer

An example of the particular involvement of UGTs in the metabolism of some NSAIDs is represented by aspirin. Aspirin is deacetylated to salicylic acid, which forms two hippuric acids (salicyluric and gentisuric) and two glucuronides. Salicylic acid accounts for 20–60% of the product while metabolites from glucuronidation are 1–42% [29]. Glucuronidations is supported by different UGT isoforms including 1A1, 1A3, 1A4, 1A6, 1A7, 1A8, 1A9, 1A10, 2B4, 2B15 AND 2B17 [30]. So, the reduction of the activity of UGTs can produce a reduction of a great part of the metabolism of the aspirin [Fig. 3].

It is also important to underline that some of the ABC members (like ABCC2 and ABCC3) drug transporters can modulate the hepatobiliary and renal transport and excretion, i.e. loss of function of these proteins can produce accumulation of reactive diclofenac glucuronides producing the effect of acute toxicity [31, 32] [Fig. 1].

#### Triptans

Triptans are used for acute treatment of migraine attacks, and their pharmacological action is based on the stimulation of serotonin receptors.

Some individual genetic traits have been associated with the variability in triptans response, as SNPs involved in transduction signal via HT1B/1D (i.e. rs5443 in the gene coding the G protein β3 subunit [33]) and SNPs in metabolic genes involved in triptans’ degradation (MAO-A and CYP1A2 and 3A4) [34]. in particular, Schürks et al. described as in a German sample rs5443 in heterozygosity (C825TC) had a positive predictive value for triptans response of 0.82 and a negative one of 0.35 [33]. Additionally, the association between genetic constitution and migraine drug response have been showed also by Christensen et al. [35]

**Fig. 4:**
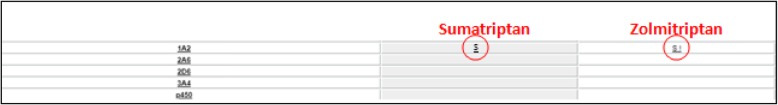
Sumatriptan and Zolmitriptan metabolic profile. From left to right, in the first column there is the list of drug metabolizing enzymes, one for each row; in the second and third columns relationship between corresponding enzyme of the row and Sumatriptan (second column) and Zolmitriptan (third column): is indicated by the symbol ‘S’ for substrate. Enzyme CYP1A2 is rimmed to emphasize their importance in these triptans’ metabolic destiny. Related page at the website http://bioinformatics.charite.de/transformer

At support of the heterogeneity in hepatic metabolism, likely due to MAO-A and CYP1A2, in different studies conducted on in migraine patients outside attacks, during attack and in healthy volunteers significant inter-individual variability was observed in the measured plasma levels of different triptans in different situations such as Cmax after oral administration of Sumatriptan [36] (metabolised by MAO-A), rather than 2 h after the administration of Zolmitriptan [37] (metabolised by CYP1A2 and MAO-A) [Fig. 4].

It is very interesting to cite the observations of Gentile et al. taking studying the CYP1A2, and in particular of the * 1F; they observed a higher frequency of -163A allele in abuser than non-abusers of drugs, hypothesizing that the -163A allele was associated to a faster degradation of the drug [34].

#### Opioids

Treatment of chronic pain is in someone entrusted to use of opioids.

This pharmacological category is even more complicated than the previous ones because, in addition to the aspects related to the enzymatic stations involved in the metabolism (mainly CYP2D6), the responsiveness to the opioid’s category is also related to the expression of dedicated mu receptors (OPRM1), which also present polymorphic alleles with differential functionality.

**Fig. 5:**
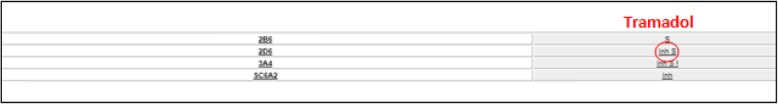
Tramadol metabolic profile. In the left column there is the list of drug metabolizing enzymes and drug transporters, one for each row; in the right column relationship between corresponding transporter or enzyme of the row and tramadol: is indicated by the symbol ‘S’ for substrate and ‘Inh’ for inhibitor. Enzyme CYP2D6 is rimmed to emphasize its importance in tramadol’s metabolic destiny. Related page at the website http://bioinformatics.charite.de/transformer

Genetic polymorphisms of CYP2D6 impact on the metabolism of this category when subjects are poor metabolizers and when are ultra-rapid metabolizers. I.e. Tramadol is a pro-drug metabolized by CYP2D6 in to its active metabolite O- desmethyltramadol [Fig. 5]. There are experimental studies that show how patients poor metabolizers had little clinical effect related to a serum concentration of the active metabolite of the lower drug compared to the dosage of tramadol administered, ultra-rapid metabolizers tend to reduced experimental pain concurrently with a wise increase in serum levels of the drug [38, 39].

In conditions of normal expression of OPRM1, poor metabolizer, not metabolizing drug, will not use it, so therapeutic effect will not be obtained. Ultra-rapid one can obtained the effect but for considerably shorter times than normal, leading to an increase in the number of administrations and doses, this could fuel an addictive mechanism towards the drug.

About the receptor, SNP identified in the region of OPRM1 leads to a substitution of aspartate for asparagine, altering N-glycosilation of the receptor protein, this influence patients’ response to therapeutic effect of opioids. Moreover, there are discordant opinions about the tendency of subjects with OPRM1 rs1799971 to make a higher use of opioids [40, 20].

#### Tricyclic antidepressants (TCAs)

Still used to treat depression, their main therapeutic use is in pain management. TCAs are mixed serotonin and norepinephrine reuptake inhibitors distinguished according to the chemical structure in tertiary amines (with a more noradrenergic effect) and secondary amines (with a more serotoninergic effect).

**Fig. 6:**
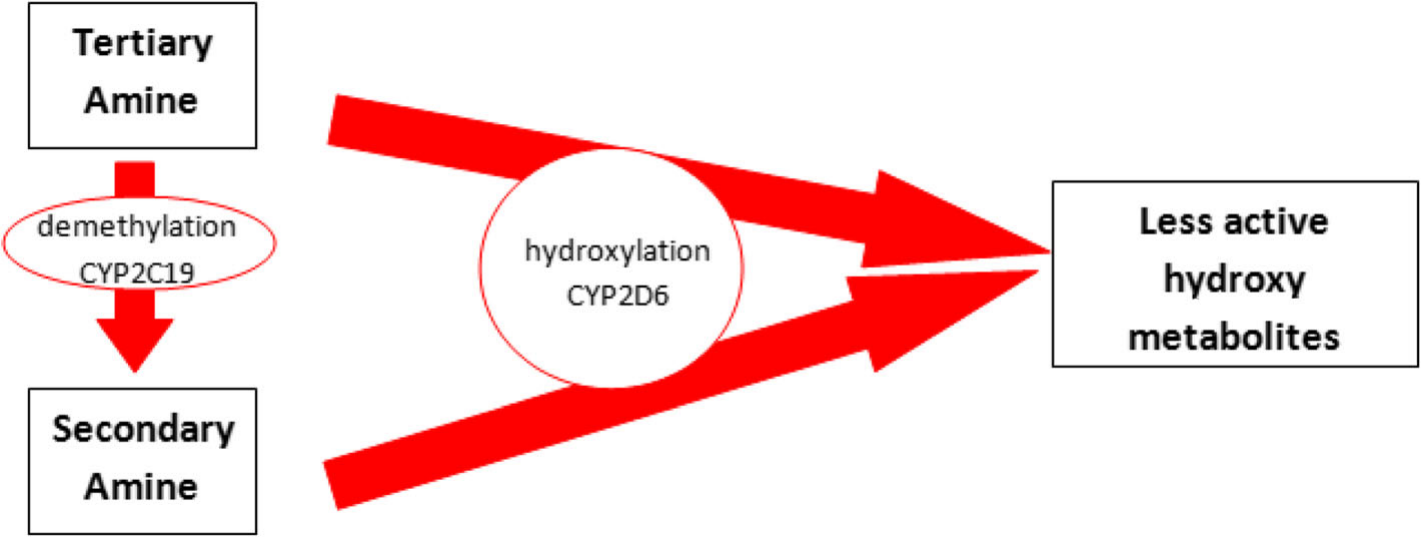
Metabolic destiny of secondary and tertiary amines. Tertiary amines trough a reaction of demethylation supported by CYP2C19 are metabolized in Secondary amines; both tertiary and secondary amines are metabolized in less active metabolites by a reaction of hydroxylation supported by CYP2D6

**Fig. 7:**
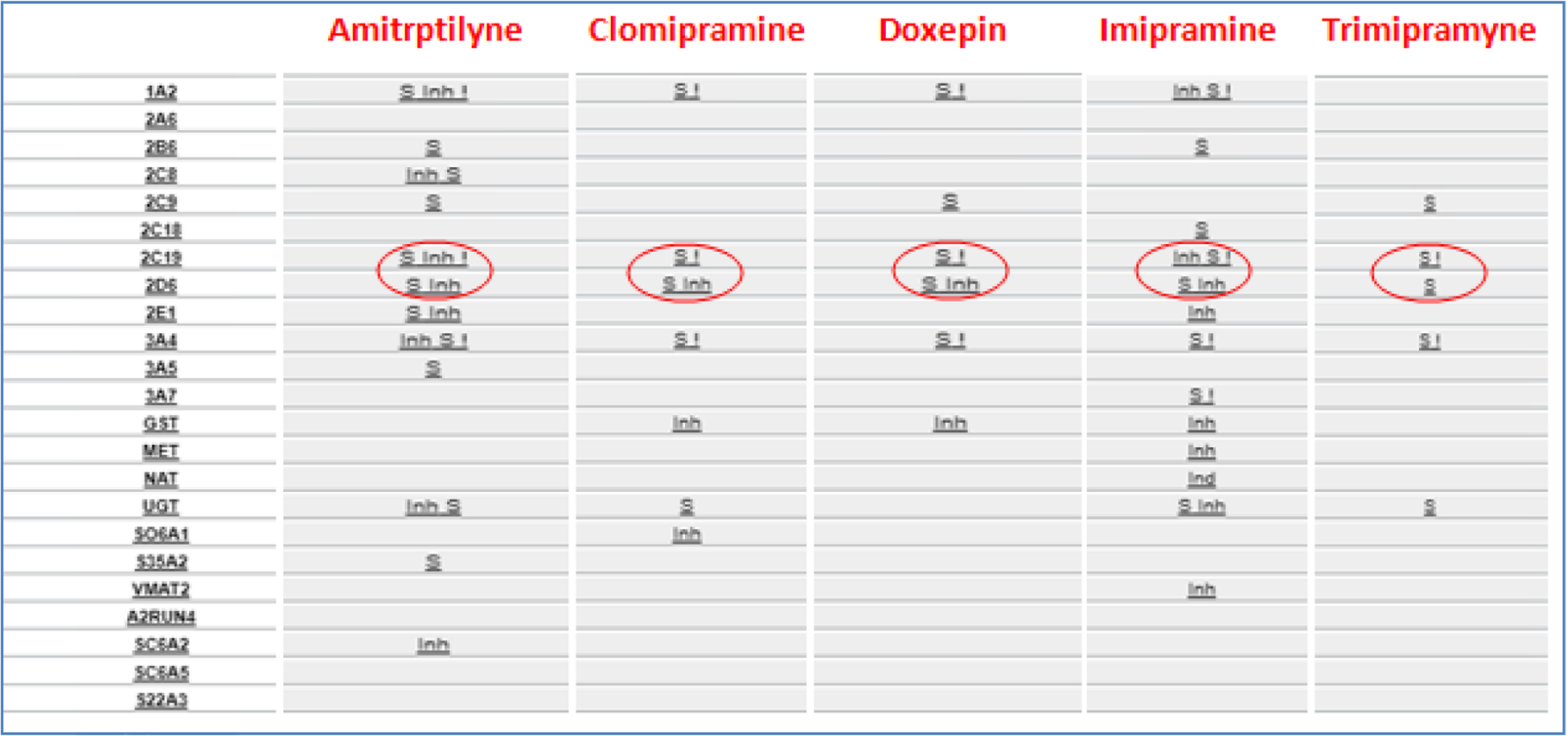
Tertiary amines metabolic profile From left to right, in the first column there is the list of drug metabolizing enzymes, one for each row; in the second, third, fourth, fifth and sixth columns relationship between corresponding enzyme of the row and different Tricyclic: is indicated by the symbol ‘S’ for substrate, ‘Inh’ for inhibitor and ‘Ind’ for inducer. Enzymes CYP2C19 and 2D6 are rimmed to emphasize their importance in these tertiary amines’ metabolic destiny. Related page at the website http://bioinformatics.charite.de/transformer

**Fig. 8:**
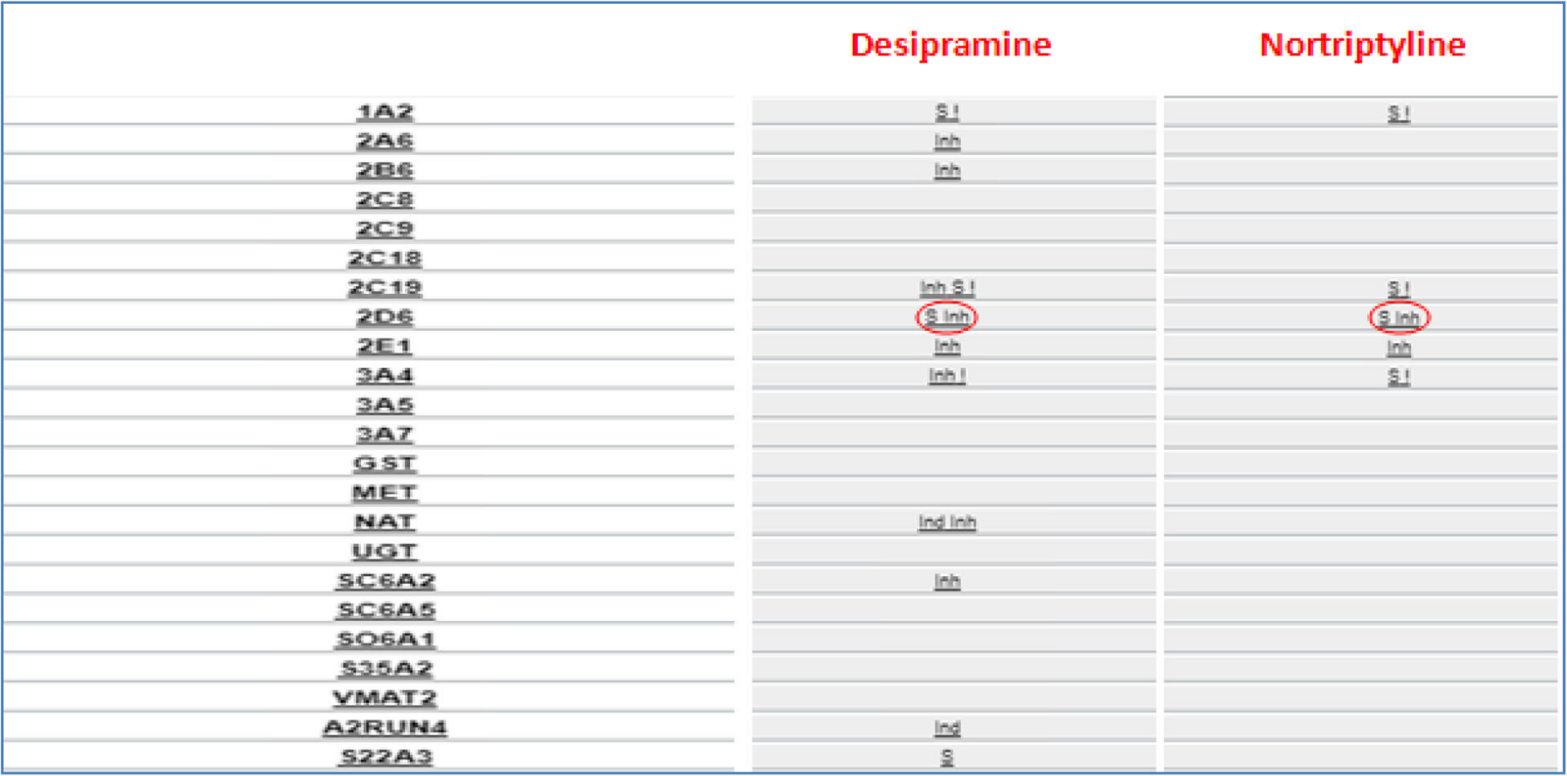
Secondary amines metabolic profile. From left to right, in the first column there is the list of drug metabolizing enzymes, one for each row; in the second and third columns relationship between corresponding enzyme of the row and different Tricyclic: is indicated by the symbol ‘S’ for substrate, ‘Inh’ for inhibitor and ‘Ind’ for inducer. Enzyme 2D6 is rimmed to emphasize their importance in these secondary amines’ metabolic destiny. Related page at the website http://bioinformatics.charite.de/transformer

By CYP2C19, tertiary amines are metabolized (demethylation) in secondary amines, both secondary and tertiary amines are metabolized to less active metabolites by CYP2D6 (hydroxylation), so it’s clear as CYP2C19 impacts the ratio of tertiary amines to secondary amines plasma concentration, but its weight on overall drug clearance is lower than CYP2D6 [Figs. 6,7,8].

It is easy to guess why often interindividual differences of plasma concentration, which are reflected in different incidence of side effect and treatment response, are registered. These differences are associated with the highly polymorphic CYP2D6 (more of 100 allelic variants and sub-variants identified) and CYP2C19 (more of 30 allelic variants and sub-variants identified). In both cases, ethnic differences were observed in the distribution of allele frequencies [41, 42]. So, knowing CYP2D6 e CYP2C19 genomic variants of a patients we could modify pharmacotherapy (type and dosage of TCAs) potentially improving clinical outcomes and reducing the rate of treatment’s failure.

There are documented cases of CYP2D6 ultrarapid patients who received large doses of tricyclic to achieve therapeutic concentrations exposing the patient himself to increased risks of adverse effects [43], likely in CYP2D6 poor patients in which a therapeutic dosage of plasma concentrations was not proportionally raised [44]. In similar situation, in both cases, therapeutic drug monitoring is strongly recommended.

In patients CYP2C19 ultrarapid, by extrapolated pharmacokinetic data, it could be said that they need increased doses of tertiary amine [45], as well as poor ones are expected to have an increase of plasma concentration if given the same dose.

Therefore, combination of traits different from extensive one, of both CYP and in the same patient could produce additive pharmacokinetic effects in tricyclic’s proprieties.

### Politherapy: the obstacles between DDI and the genetic trait

According with how until now explained and in consideration of the fact that, as reported by the studies of Ferrari et al. [19], it’s common practice to treat migraine with multiple types of medications, the limit of patient’s genetic is compounded by interaction that can settle down between each drug. In fact, it must be also considered how the risk of toxicity and inefficacy of a polytherapeutic regime is partly attributable to the mechanism for which the pharmacological effect of a drug varies due to the simultaneous biological action of an additional drug on the metabolic stations used for the metabolism of the first drug, but equally and with reversed roles applies to the second drug too: the efficacy or possible toxicity of a pharmacological cocktail is partly attributable to the drug-drug interactions (DDIs) that are established between the various drugs in therapy [46]. It’s clear that the more drugs are present into the therapeutic regimen, the more DDIs need to be considered. Therefore, it is evident that the multiple comorbidities that frequently occur in specific subsets of patients with migraine (cardiovascular, cerebrovascular, psychiatric and musculoskeletal) [47–49] and which require the introduction of other drugs into therapy, further complicate the situation.

Moreover, as previously demonstrated, genetic trait of patient impacts further on the efficacy and toxicity of a drug. When a therapy is based on more than one drug, the therapist has to consider the situation in all its completeness. Unfavourable drug-drug and/or drug-drug-genome interaction can represent greats risk factor in the development of adverse drug reaction (ADRs), related to deficient therapeutic effect or toxicity [50]. And in these ADRs the possible real motivation of many of the therapeutic failures that aggravate already complicated clinical pictures is to be found, they maintain the pathogenetic processes and induce the chronification of the pathology.

**Fig. 9:**
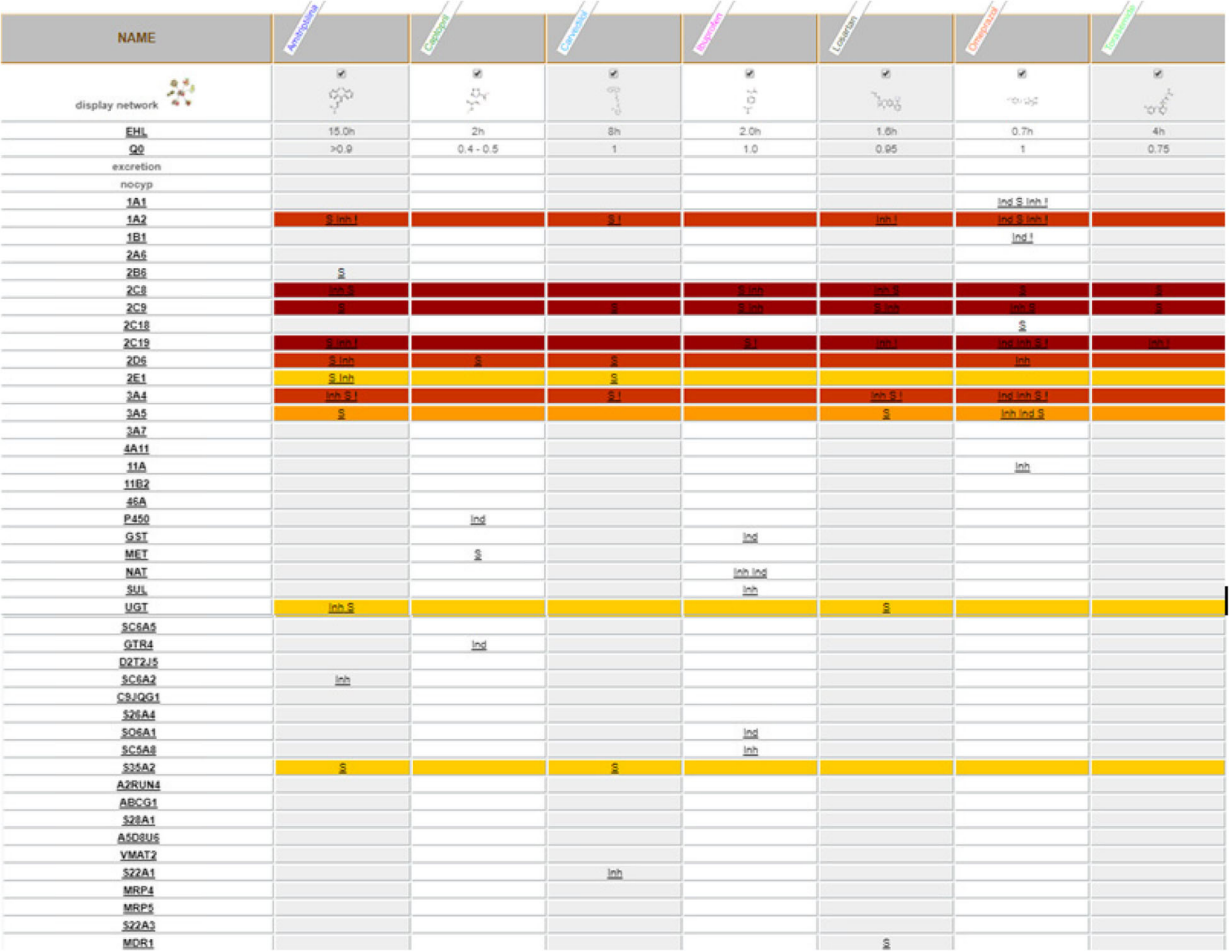
Drug-drug interaction involved in a polytherapy for hypertension, prophylactic therapy for chronic migraine and episodes of acute attacks. From left to right, in the first column there is the list of drug metabolizing enzymes, one for each row, each following column represent a drug the relationship between a drug and an enzyme/transporter is indicated by the symbol ‘S’ for substrate, ‘Inh’ for inhibitor and ‘Ind’ for inducer. The colours of different rows indicate the increase in metabolic pressure passing by the various colours ranging from yellow to orange, to red, to dark red. Related page at the website http://bioinformatics.charite.de/transformer

For the explanatory purpose of the above-mentioned, let consider the plausible situation of a patient suffering from arterial hypertension and chronic migraine. The patient in question is treated for the arterial hypertension with a sartan (Losartan), a β-blocker (Carvedilol), an Ace-inhibitor (Captopril), a diuretic (Torasemide); for the prophylactic treatment of migraine, he takes a tricyclic (Amitriptyline); during migraine attacks he uses an NSAID (Ibuprofen); to complete this therapeutic regimen employs a PPI (Omeprazol) [Fig. 9].

Without information about the genetic profile of the patient, it is possible to state that the therapeutic regimen is not the best under the metabolic point of view. In fact, it’s evident as drugs present in the proposed regimen impact in a different way (as substrate, inhibitors and inductors) on differently enzymatic stations variously important for the drug category considered.

If to that an unfavourable genetic trait is added, as in the example patient (poor metabolizer for CYP 2D6 and CYP2C19, and with reduction of activity of CYP 2C9), it’s clear that therapeutic regimen is not well thought out. Probably Amitriptyline will not work (it’s a tertiary amine that need to be transformed by CYP2C19 in secondary to be then hydrossilated by 2D6), the same for Captopril (substrate of CYP2D6), Carvedilol (substrate of CYP2C9 and 2D6), Ibuprofen (substrate and inhibitors of CYP2C9), Losartan (inhibitor and substrate of CYP2C9, inhibitor of CYP2C19), Omeprazol (primary substrate, inductor and inhibitor of CYP2C19, but moreover substrate and inhibitor of CYP2C9 and inhibitor of 2D6) and Torasemide (substrate of CYP2C9 and inhibitor of 2C19). These only citing the enzymatic stations that would show a reduced activity on the basis of the genetic trait.

**Fig. 10:**
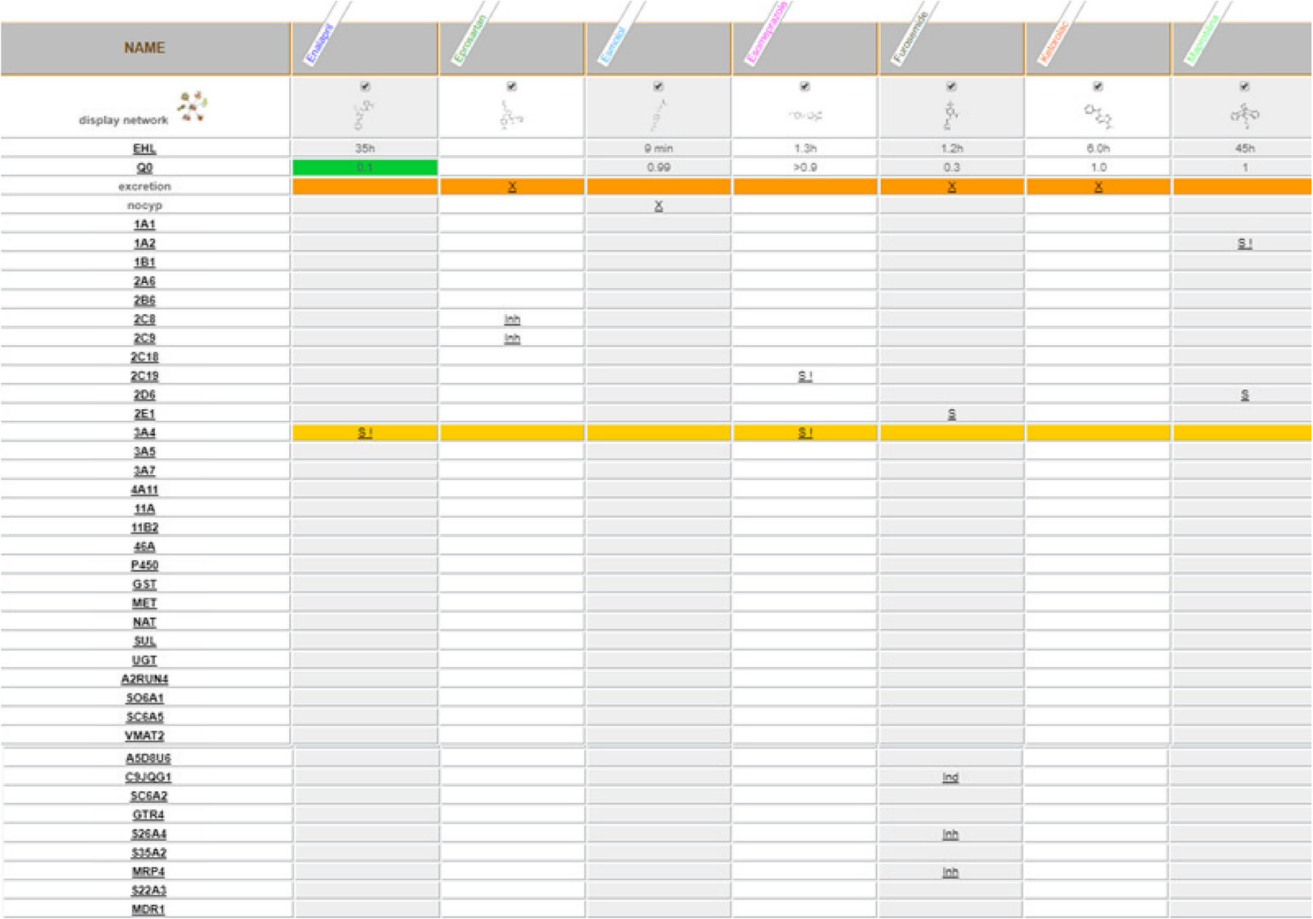
Drug-drug interaction involved in optimized polytherapy for hypertension, prophylactic therapy for chronic migraine and episodes of acute attacks optimization of previous therapy. From left to right, in the first column there is the list of drug metabolizing enzymes, one for each row, each following column represent a drug the relationship between a drug and an enzyme/transporter is indicated by the symbol ‘S’ for substrate, ‘Inh’ for inhibitor and ‘Ind’ for inducer. The colours of different rows indicate the increase in metabolic pressure passing by the various colours ranging from yellow to orange, to red, to dark red. X = link to related scientific articles about the items in the first column accessible through the related page at the website http://bioinformatics.charite.de/transformer

A therapeutic approach based on the personalized medicine allows to remedy similar situation by setting from the beginning a therapy based on drugs metabolically non-interfering with each other and with the functional biochemical profile of the patient, or alternatively, in the case of already established therapies, adjusting the shot making the therapeutic regime more effective and avoiding the ADRs that can develop due to unfavourable drug-drug and/or drug-drug-genome interactions. In referring to previous example, the therapeutic regimen could be optimized choosing drugs compatible both with biochemical profile of the patient and with his clinical necessity, for example selecting as sartan Eprosartan (that differently from Losartan is only inhibitor, but not substrate of CYP2C9, ant it is not inhibitor of CYP2C19), as β-blocker Esmolol (that differently from Carvedilol not is substrate of CYP2C9 and CYP2D6), as Ace-inhibitor Enalapril (that differently from Captopril is not substrate of CYP2D6), as diuretic Furosemide (that differently from Torasemide not is substrate of CYP2C9 and inhibitor of CYP2C19), as tricyclic Maprotyline (that differently from Amitriptyline it is only substrate but not inhibitor of CYP2D6 and is not substrate of CYP2C19), as PPI Esomeprazole (that differently from Omeprazol is only inhibitor but not substrate of CYP2C19 and is not substrate and inhibitor of CYP2C9 and inhibitor of 2D6), at last in case of acute attacks as NSAIDs Ketorolac (that differently from Ibuprofen is not substrate and inhibitors of CYP2C9). Moreover, in this way, drug-drug interactions that can be unfavourable on other metabolic stations are drastically reduced. [Fig. 10].

### New therapeutic perspectives

The possibility of a personalized pharmacological polypharmacy, calibrated on the patient’s functional biochemical abilities and on the further therapeutic necessities dictated by the comorbidities of the same, seems to contrast with some biotechnological drugs, albeit with the limit of being mostly still under study. It’s a matter of monoclonal antibodies, antagonist molecules and agonist molecules crucial in migraine mechanism. These not having a metabolic destiny, or rather not being subjected to enzymatic transformations or substrates of membrane transporters, allow to bypass the obstacles dictated by different functional biochemical settings of each individual patient and by the metabolically unfavourable drug interactions, common in the polytherapies.

One of the pathogenetic mechanisms under study for the structuring of the drugs in question is represented by the activation of trigeminal neurons which involves the release of some neuropeptides (CGRP substance P, PACAP and nitric oxide). These cause at the peripheral level vasodilatation of the meningeal blood vessels and reflex stimulation of the parasympathetic system [51]. In particular, to date, therapeutic drugs interfere with the vasodilatory mechanism induced by the CGRP are in use and object of study. Regarding the use in the acute phase, two categories of drugs have been designed (Gepants and Ditans), whereas monoclonal antibodies against CGPR have been developed for prophylactic purposes.

#### Gepants

Gepants are non –peptide CGRP able to reduce the activity of the trigeminal- vascular system. Their effectiveness is similar to the triptans one, but differently from triptans not inducing vasoconstriction, gepants have no side effect related to this event. Moreover, they show a prolonged effect of action compared to the triptans [52].

Among these, olcegepant (BIBN4096BS) is the first neuropeptide antagonist of CGRP receptor used with success since 10 years [53]. This drug binds a part of the CGRP receptor (RAMP1), competing with endogenus CGRP [54]. Unfortunately, the bioavailability is reduced by oral abministration because this drug has a poor penetration across the Blood-brain barrier (BBB), in fact it proves effective after intravenous administration, this constitutes an obstacle to the common use from migraineurs [55].

Telcagepant (MK - 0974) is the oral CGRP receptor antagonist developed following in the footsteps of the olcegepant. It is rapidly absorbed, with a Tmax of 1.5 h and terminal half-life 6 h [56], it proves effective in treating migraine associated symptoms, such as photophobia, phonophobia and nausea. But the most important side effect is a hepatotoxicity that may be dose- and time-dependent in consideration to an observed increase in transaminases [55].

Ubrogepant (MK-1602) and Rimegepant (BMS-927711) actually at phase III of study, represents the latest gepants object of study, but there are currently no definitive data regarding efficacy, bioavailability of side effects of such drugs [57].

#### Ditans

Ditans are agonist of 5-HTR selective for the type 1F, this one decreases the release of excitatory transmitters and CGRP in a trigeminal-vascular system. Differently from triptans, that bind to the 5HT _1B_ e 5HT _1D_ receptors, they do not induce peripheral vasoconstriction despite having a similar therapeutic efficacy on the migraine. So, they are better tolerated and with less contraindications related to the peripheral vasoconstriction [58]. The most used today is Lasmiditan, that was shown to be efficacious and well tolerated in the treatment of acute migraine in patients with a high level of cardiovascular risk factors [59].

#### CGRP monoclonal antibodies

The use with a prophylaxis purpose is supported by their lower onset of action and much longer half-life, differently from CGRP receptor antagonist. Compared to other drugs used in prophylaxis CGRP monoclonal antibodies might be administrated less frequently, in fact previous drugs (like triptans) are recommended orally one to three times daily, antibodies one up to once a month [60]. Compared from CGRP’s receptor antagonists these monoclonal antibodies are highly selective, this avoids the reported toxic effects of CGRP’s receptor antagonists. Moreover, different studies, as early clinical trials, have also shown that humanized monoclonal antibodies against CGRP have proven successful in reducing the frequency of migraine headaches as a preventative therapeutic [61]. However, there are polymorphism in the CGRP receptor pathway, which have been investigated, that increase the risk of migraine evolution into the complication of medication oversue [62]. We also have to mention a negative study on this matter revealing that polymorphism in CGRP pathaways might be the signal of differences between CGRP mAB responders vs. non-responders [63]. The side effects of this monoclonal antibodies are to be found in the protective role of CGRP. This is able to counteract the development of hypertension, because it has a direct action on smooth muscle cells in the vessel wall, particularly marked at the microvascular level, to which it is attributed the establishment of peripheral resistance and so of the blood pressure. In the same way, having CGRP an vasodilatory effect, the use of this monoclonal antibodies induces a reduction of CGRP’s in cardio-protective mechanisms during ischemia [64]. The unique drug directed against the receptor is Erenumab, the other ones (Galcanezumab, Fremanezumab and Eptinezumab) are directed against CGRP.

Erenumab is a human immunoglobulin G2 monoclonal antibody designed specifically to bind and antagonize the calcitonin gene-related peptide receptor (CGRPR). The most common side effects of erenumab include pain, redness, or swelling at the injection site, and constipation.

Galcanezumab is a fully humanized monoclonal antibody against human calcitonin gene-related peptide (CGRP), is administered as a subcutaneous injection. There are clinical evidence that shown a significant reduction in the mean number of migraine headache days and a drug’s good tolerability profile [65]. The most commonly reported adverse events are headache, nasopharyngitis, hematuria, dermatitis, diarrhea, toothache, and increased alanine aminotransferase (ALT) [61].

Fremanezumab is a genetically engineered humanized monoclonal antibody against human calcitonin gene-related peptide (CGRP) [66]. Ongoing clinical trials for the agent are directed to people with episodic and chronic migraine as well as cluster headaches. It is administrated in a monthly subcutaneous injection [67]. The most commonly reported adverse events included injection site erythema, injection site induration, diarrhea, anxiety, and depression [68].

Eptinezumab is a fully humanized IgG1 antibody manufactured using yeast [64]. It is currently in clinical trials for preventing migraine attacks. It has been specifically designed to bind to both alpha and beta forms of the human calcitonin gene-related peptide (CGRP). The most frequent adverse events include upper respiratory tract infection, urinary tract infection, fatigue, back pain, arthralgia, and nausea and vomiting [69].

## Conclusions

A personalized approach for setting the therapies that every patient needs, dictated by the evaluation of the comorbidities and the functional biochemical structure of the same, represents a goal in the therapeutic field by reducing the possibility of establishing side effects related to therapies that affect the clinical course of each patient. The new biotechnological drugs currently being studied could represent a valid alternative that needs to be further refined to date, with the aim of reducing the already highlighted limitations of the same correlated to the contraindications linked to the comorbidities and to the adverse effects recorded.

## Abbreviations

ACE: Angiotensin-converting enzyme
ADR: Adverse drug reaction
ALT: Alanine aminotransferase
BBB: Blood-brain barrier
CGRP: Calcitonin gene related peptide
CH: Chronic headache
DDI: Drug – drug interaction
EDNRA: Endothelin type A receptor
GWAS: Genome-wide association studies
MA: Migraine with aura
MAO-A: Monoamine oxidase A
MHTFR: Methylenetetrahydrofolate
MO: Migraine without aura
MOH: Medications overuse headache
NOS3: End othelial nitric oxide synthase type 3
NOTCH3: Neurogenic locus notch homolog protein 3
NSAID: Nonsteroidal anti-inflammatory drugs
OPRM1: Opioid receptor mu 1
PPI: Proton-pump inhibitor
SNP: Single nucleotide polymorphism
TCA: Tricyclic Antidepressant
TGFB2: β-2 transforming growth factor
TGFB2R: β-2 transforming growth factor receptor
VIP: Vasoactive intestinal peptide
